# Elevated incidence of somatic mutations at prevalent genetic sites

**DOI:** 10.1093/bib/bbae065

**Published:** 2024-02-28

**Authors:** Mengyao Wang, Shuai Cheng Li, Bairong Shen

**Affiliations:** Department of Computer Science, City University of Hong Kong, 83 Tat Chee Ave, Kowloon Tong, Hong Kong, China; Laboratory of Gastrointestinal Cancer (Fujian Medical University), Ministry of Education, Fuzhou, China; Department of Computer Science, City University of Hong Kong, 83 Tat Chee Ave, Kowloon Tong, Hong Kong, China; Institutes for Systems Genetics, Frontiers Science Center for Disease-Related Molecular Network, West China Hospital, Sichuan University, Chengdu, Sichuan, China; Joint Laboratory of Artificial Intelligence for Critical Care Medicine, Department of Critical Care Medicine and Institutes for Systems Genetics, Frontiers Science Center for Disease-related Molecular Network, West China Hospital, Sichuan University, 610212, Chengdu, China

**Keywords:** common loci, mutation incidence, TCGA somatic mutations, aging

## Abstract

The *common* loci represent a distinct set of the human genome sites that harbor genetic variants found in at least 1% of the population. Small somatic mutations occur at the common loci and non-common loci, i.e. csmVariants and ncsmVariants, are presumed with similar probabilities. However, our work revealed that within the coding region, common loci constituted only 1.03% of all loci, yet they accounted for 5.14% of TCGA somatic mutations. Furthermore, the small somatic mutation incidence rate at these common loci was 2.7 times that observed in the non-common. Notably, the csmVariants exhibited an impressive recurrent rate of 36.14%, which was 2.59 times of the ncsmVariants. The C-to-T transition at the CpG sites accounted for 32.41% of the csmVariants, which was 2.93 times for the ncsmVariants. Interestingly, the aging-related mutational signature contributed to 13.87% of the csmVariants, 5.5 times that of ncsmVariants. Moreover, 35.93% of the csmVariants contexts exhibited palindromic features, outperforming ncsmVariant contexts by 1.84 times. Notably, cancer patients with higher csmVariants rates had better progression-free survival. Furthermore, cancer patients with high-frequency csmVariants enriched with mismatch repair deficiency were also associated with better progression-free survival. The accumulation of csmVariants during cancerogenesis is a complex process influenced by various factors. These include the presence of a substantial percentage of palindromic sequences at csmVariants sites, the impact of aging and DNA mismatch repair deficiency. Together, these factors contribute to the higher somatic mutation incidence rates of common loci and the overall accumulation of csmVariants in cancer development.

## INTRODUCTION

As the common loci represent a specific subset of genomics sites within the human genome containing genetic variants observed in at least 1% of the population, investigating potential differences in somatic mutations between common loci and non-common loci is a significant research endeavor. Single nucleotide polymorphisms (SNPs) are pervasive across the human genome and have been identified in significant numbers [1]. To date, the single nucleotide polymorphism database (dbSNP) has amassed approximately 38 million genetic variants, including SNPs and small insertions and deletions, classified as common [2]. These common small variants exhibit a minor allele frequency greater than 1% and account for 6.72% of total small variants recorded by dbSNP. These common small variants are less likely to be linked to severe genetic diseases, owing to the impacts of natural selection [3].

The nonuniform distribution of SNP density across the genome, influenced by factors like selection pressure, recombination rates and mutation rates, results in varying mutation rates. SNPs, particularly those in noncoding regions, impact disease development and clinical phenotypes by influencing various cellular processes [4–6]. Mutation rates vary throughout the genome, and CpG dinucleotides exhibit a higher mutation rate due to cytosine deamination and deficient DNA mismatch repair [7–11]. DNA palindromes, sequences reading the same forward and backward, contribute to genomic instability, potentially inhibiting DNA mismatch repair [12, 13]. Somatic mutations, distinctive genetic alterations exclusive to the body’s somatic cells, play a pivotal role in disease development, notably in cancer. Serving as fundamental elements driving carcinogenesis and disease progression, these mutations can arise spontaneously or due to environmental factors like radiation or chemicals [14]. The somatic mutation rate is notably elevated in intergenic regions compared with other functional elements of the genome [15].

While a considerable number of cancer-associated variants are cataloged in dbSNP, only nine were identified by the 1000 Genomes Project among the submitted genetic variants [16]. Remarkably, many cancer genome studies overlook somatic mutations, particularly common small variants prevalent in population genomes. Commonly perceived as unrelated to cancer, these small variants are often deprioritized in cancer-related analyses [16]. Given the high incidence rate of multiple common small variant loci in cancer, it becomes imperative to comprehensively investigate somatic mutations occurring at these common loci during cancer genome research.

**Figure 1:**
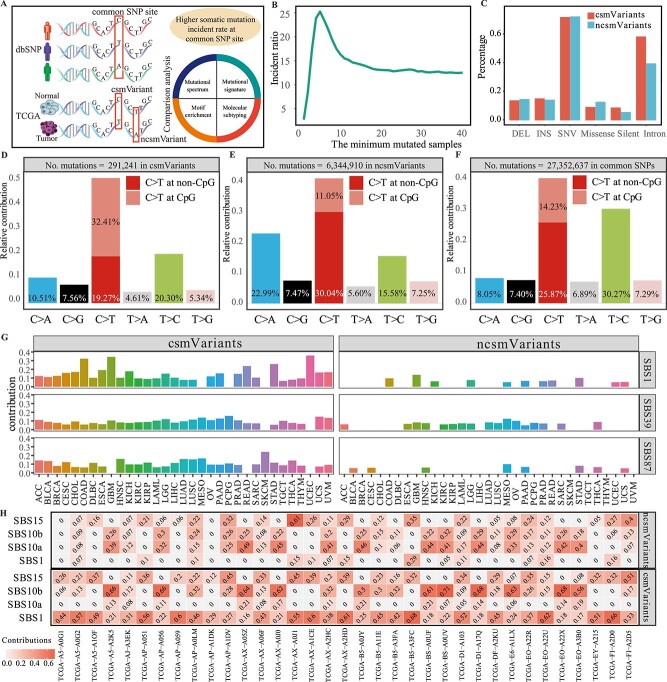
**The characters of csmVariants compared with ncsmVariants.** (A) The overview of csmVariants comparison study. (B) The distribution of somatic mutation incidence ratio of common loci to non-common loci in the coding region. The x-axis refers to the minimum mutation number (mutated samples). The y-axis denotes the ratio of somatic mutation incidence rate to that of non-common loci. The curve displayed the incidence ratio among mutation loci that mutated in more than n samples (the minimum mutation number is n). The incidence ratio under a minimum mutation number of 2 meant that the incidence rate of somatic mutations mutated more than once at common loci compared with that at non-common loci. (C) Comparing the percentage of different mutation types in csmVariants and ncsmVariants datasets. We also displayed the relative contributions of different substitution types in csmVariants (D), ncsmVariant (E) and common SNPs set (F). (G) We displayed the contributions of three SBS mutational signatures that had significant differences in csmVariants and ncsmVariants. (H) Comparing the contributions of SBS1, SBS10a, SBS10b and SBS15 in 35 hyper-mutated TCGA-UCEC samples with over 500 csmVariants.

In this study, small somatic mutations that occur at common small variants loci were defined as common somatic mutated Variants (csmVariants, Figure 1A). The small somatic mutations include single nucleotide variants (SNVs), insertion (INS) and deletion (DEL). The non-common loci are genome regions excluding the common loci. The ncsmVariants are small somatic mutations occurring at non-common loci.

5.51% of TCGA small somatic mutations are identified as csmVariants, and 36.14% of them are recurrent. csmVariants are characterized predominantly by C-to-T mutations at CpG sites, and palindromic sequences are prevalent in the context of csmVariants. The high somatic mutation incidence rate of common loci reflects the prevalence of DNA substitution at the common loci, which could provide a novel perspective for unraveling the accumulation of somatic mutations during cancerogenesis.

## MATERIALS AND METHODS

### Identifying csmVariants from TCGA somatic mutation dataset

To explore the small somatic mutations that occurred at the common loci or non-common loci, we divided the TCGA somatic mutations into two groups: csmVariants and ncsmVariants. First, we retrieved the common genetic variants dataset with a minor allele frequency of at least 1% in the 1000 Genomes population from NCBI (https://ftp.ncbi.nih.gov/snp/organisms/human_9606/VCF/common_all_20180418.vcf.gz). Then, we collected the TCGA small somatic mutations data from the Genomic Data Commons Data Portal (https://portal.gdc.cancer.gov/), which comprised 10 044 samples across 33 types of cancer. The TCGA project has sequenced more than 11 000 cancer samples and provides the publicly accessible somatic mutation dataset online. We removed somatic mutations with no less than three reads support in matched normal tissue, or lower than 5x sequencing depth in the tumor, or lower than 0.05 variant allele frequency in the tumor. We used the common small variant sites to identify csmVariants in the TCGA somatic mutations dataset. Finally, we found 341 709 csmVariants among 10 044 tumor samples. We downloaded the regulatory features (Promoter, Enhancer, CTCF binding sit and Open chromatin region) from the Ensembl Regulation (https://ftp.ensembl.org/pub/current_regulation/homo_sapiens/homo_sapiens.GRCh38.Regulatory_Build.regulatory_features.20221007.gff.gz), which provides the computational annotation of regulatory features in HG38 reference. Then we selected the csmVariants located in the regulatory feature regions and identified the significantly mutated noncoding functional csmVariants in the TCGA dataset.

The common SNPs set hereinafter refer to the downloaded common genetic variants dataset, and the TCGA mutation dataset refers to the downloaded TCGA somatic mutations.

### Deciphering mutational signatures

To decipher the mutational signatures and visualize the mutational profiles, we employed the R package ‘sigminer’ [17] (v2.1.9). First, we used this package to generate a matrix composed of the count of 96 trinucleotide contexts for the csmVariants or ncsmVariants of each sample. Then, we merged the matrices by cancer types to generate a new 96x33 matrix, with the column names being the 96 trinucleotide contexts and the row names being the 33 cancer types. We decomposed the 96x33 matrix into known mutation signatures of the Catalogue Of Somatic Mutations In Cancer (COSMIC) using the signature decomposition function of ‘sigminer’, which can determine the contribution of each mutational signature to each cancer type. For the selected TCGA-UCEC samples, we decomposed the contribution of each mutational signature to the csmVariants or ncsmVariants for each sample, separately.

### Molecular subtyping with frequently mutated csmVariants

We selected 11 frequently mutated csmVariants that had higher than 10% mutation frequency in specific cancer types and divided these patients into two groups by whether they carried this csmVariant or not. With the TCGA clinical data downloaded from the integrated TCGA Pan-Cancer Clinical Data Resource [18], we performed Kaplan–Meier survival analysis with the R package ‘survminer’ (v0.4.9). To discern the prognostic impact of the csmVariant rate, we performed multivariate Cox regression analysis, adjusting for age, gender and tumor stage. We utilized a maximum-likelihood dN/dS method to identify the significantly mutated genes (SMGs) in different cancer groups [19]. Moreover, for each cancer group, we decomposed and compared the contribution of each COSMIC mutational signature, shedding light on the intricate mutational landscape underlying these cancers.

### Motif enrichment in csmVariant contexts

We meticulously extracted the sequence context surrounding each csmVariant, capturing 10 flanking bases both 5’ and 3’ to the variant. Subsequently, we utilized STREME [20] to discover motifs that were enriched in the csmVariant context sequences. This analysis facilitated a deeper understanding of the specific nucleotide patterns contributing to the enrichment observed in csmVariants. We then used SEA (MEME-V5.5.0) [21] to examine the distribution of the 35 significantly enriched motifs in csmVariants of each cancer type, as well as within common SNPs set and ncsmVariants. SEA’s capabilities enabled us to measure the enrichment of these identified motifs effectively. It is crucial to note that the motifs’ letters may represent multiple nucleotides based on the position weight matrix, denoting: ‘N’: A/T/C/G; ‘D’: A/G/T; ‘R’: A/G; ‘Y’: C/T; ‘H’: A/C/T; ‘M’: C/G.

We applied IUPACpal [22] to identify the palindromic sequences from the csmVariants context with parameters set to be ‘-m 4 -M 20 -g 5 -x 1’, and we excluded palindromic sequences that are 4bp in length and contain one mismatch. If the csmVariants were not located on the stem or loop region of the identified palindromic structure, we also eliminated these palindromic sequences.

## RESULTS AND DISCUSSIONS

### Higher somatic mutation incidence rate of common loci than non-common loci

The somatic mutation incidence rate of common loci was 2.7 times that of non-common loci. Approximately 5.51% TCGA somatic mutations were identified as csmVariants. In the coding regions, only 1.03% of loci were categorized as common; nonetheless, 5.14% of TCGA somatic mutations were observed at these common loci, leaving the remaining 94.86% to occur in the non-common loci. This suggested that somatic mutations were more prone to common loci than non-common loci. We defined the somatic mutation incidence rate of one region as the number of mutated loci divided by the total loci counts in this region. For the coding region, the somatic mutation incidence rate at common loci was 16.48%, while the incidence rate of non-common was 6.06%. The somatic mutation incidence ratio of common loci versus non-common loci was 2.7. Moreover, this ratio increases when focusing on somatic mutations with higher mutation frequencies (Figure 1B, Supplementary Table S1). The variant allele frequency distribution of csmVariants resembled that of ncsmVariants (Supplementary Figure S1B, C). We defined the recurrent somatic mutation rate as the percentage of mutation sites that mutated more than once divided by the total number of mutation sites. Recurrent somatic mutation rates of csmVariants and ncsmVariants were 36.14% and 13.97%, respectively (Supplementary Table S2). The elevated recurrence of somatic mutations in csmVariants also indicated that these common loci were more prone to somatic mutation than non-common loci.

The proportion of SNV, INS and DEL in csmVariants and TCGA mutation datasets were similar, and SNV was approximately five times as high as INS or DEL in csmVariants and TCGA mutation datasets (Figure 1C, Supplementary Table S3). Regarding the various types of mutation, ‘Intron’, ‘Missense’ and ‘Silent’ comprised 57.90%, 8.60% and 8.19% of csmVariants, and 39.10%, 12.23% and 5.04% of ncsmVariants, respectively (Figure 1C, Supplementary Table S3). It is well established that different genomic regions have distinct rates of somatic mutation [23]. Remarkably, the proportion difference of csmVariants between the intronic region and the missense exonic sites was even larger than the proportion difference of ncsmVariants (6.7 fold versus 3.2 fold; 57.90% versus 8.60% in csmVariants, 39.10% versus 12.23% in ncsmVariants). The csmVariants call rates (the csmVariants call rate of one mutation type is the count of csmVariants divided by the count of TGCA somatic mutations marked with same mutation type) for ‘Intron’, ‘Missense’ and ‘Silent’ mutations were 8.16%, 3.88% and 8.96%, respectively. Notably, the csmVariants call rate for ‘Missense’ was lower than that of ‘Intron’ or ‘Silent’ mutation types.

Why is the somatic mutation incidence rate higher in csmVariants than in ncsmVariants? In the subsequent section, we will conduct a comparative analysis between csmVariants and ncsmVariants to elucidate potential molecular mechanisms.

### csmVariants are characterized predominantly by C-to-T transitions at CpG sites

C-to-T transitions at CpG sites contributed to 32.41% of csmVariants, 2.93 times to ncsmVariants. The distribution of ‘C-to-T’, ‘T-to-C’, ‘C-to-A’, ‘C-to-G’, ‘T-to-G’ and ‘T-to-A’ single-base substitutions (SBSs) was 51.68%, 20.30%, 10.51%, 7.56%, 5.34% and 4.61% (Figure 1D, Supplementary Table S4). Additionally, the frequency of C-to-T substitutions at CpG sites surpassed that of non-CpG C-to-T transitions, with CpG C-to-T transitions accounting for 32.41% of the total mutations, while non-CpG C-to-T transitions only account for 19.27%. The C-to-T transitions at CpG sites of the csmVariants (32.41%) were more frequent than the ncsmVariants (11.05%) and common SNPs set (14.23%, Figure 1E, F). The common SNPs set referred to the common SNPs recorded by dbSNP. The mutational profile of the 96 trinucleotides of the csmVariants was primarily characterized by NCG C-to-T mutation type (Supplementary Figure S2A), which was similar to the COSMIC SBS1 signature (cosine similarity: 85.98%).

Furthermore, there were 755 doublet base substitutions (DBSs) at the csmVariants site, with the ‘TG-to-CA’ mutation type having the highest proportion (16.59%). In contrast, in the ncsmVariants, ‘CC-to-TT’ had the highest frequency (30.78%), while the frequency of ‘TG-to-CA’ is only 4.10% (Supplementary Figure S2B). In terms of small insertions and deletions (ID) at the csmVariants site, there were 49,713 occurrences. Among these, ‘1:D:C’ (delete 1 C from a stretch of 1 C, 8.44% versus 2.16 %), ‘1:I:C’(insert 1 C with no neighboring C, 6.89% versus 4.06%) and ‘1:I:T’(insert 1 T with no neighboring T, 12.3% versus 5.7%) ID types had a higher frequency in the csmVariants than in the ncsmVariants (Supplementary Figure S2C).

### Aging-associated signature SBS1 exhibits the highest level of activity in csmVariants

The aging-associated mutational signature contributed to 13.87% of the observed csmVariants. We conducted mutational signature analyses on both csmVariants and ncsmVariants to determine the contribution of SBS, DBS and ID signatures recorded by COSMIC to each cancer type. SBS1 (mean value: 13.87% versus 2.52%, t.test $P-value = 2.1e-08$), SBS39 (8.92% versus 3.91%, t.test $P-value = 1.11e-06$) and SBS87 (10.02% versus 1.62%, t.test $P-value = 3.06e-11$) had significantly higher contributions to csmVariants than the ncsmVariants (Figure 1G, Supplementary Table S5). SBS1 is an endogenous signature characterized predominantly by C-to-T mutations at CpG sites, which is initiated by the spontaneous or enzymatic deamination of 5-methylcytosine to thymine. On the other hand, SBS87, associated with thiopurine chemotherapy treatment, is predominantly characterized by substitutions at CpG sites. We selected 35 hyper-mutated TCGA Uterine Corpus Endometrial Carcinoma (UCEC) samples with over 500 csmVariants and compared the contribution of each mutational signature in csmVariants and ncsmVariants. SBS1 also had a significantly higher contribution to csmVariants than the ncsmVariants (mean value: 39.21% versus 4.55%, t.test $P-value = 2.1e-08$, Figure 1H).

Similarly, Signature DBS7, characterized predominantly by TT-to-NN double mutations, was significantly enriched in csmVariants compared with the ncsmVariants (mean value: 25.12% versus 14.05%, t.test $P-value = 8.1e-06$, Supplementary Figure S3A, Supplementary Table S6). Notably, DBS7 was related to defective MMR. The MMR deficiency also includes failure to detect and remove mismatches resulting from CpG deamination, leading to C-to-T transitions at CpG sites [24]. Additionally, signature ID9, which was predominantly characterized by $<5$ bp deletions, was significantly enriched in csmVariants compared with the ncsmVariants (mean value: 20.54% versus 2.85%, t.test $P-value < 2.2e-16$, Supplementary Figure S3B, Supplementary Table S7).

### Enrichment of Palindromic sequences in csmVariants nearby context

**Figure 2:**
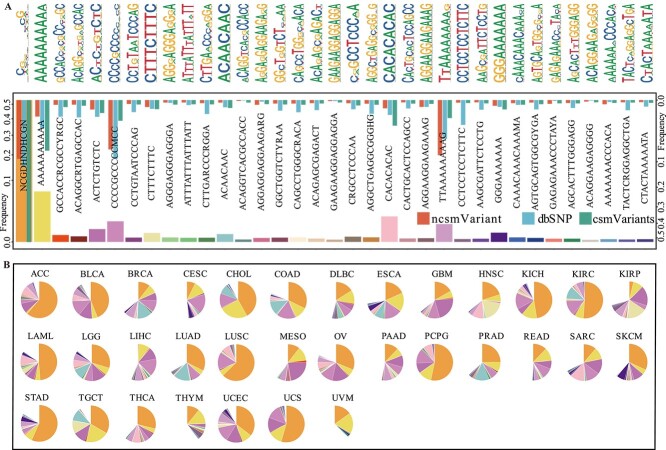
**Overview of the motifs in csmVariants context.** (A) The upper panel displayed the sequence logo of the 33 motifs. The lower panel displayed the frequency of 33 motifs in csmVariants, common SNPs and ncsmVariants. (B) The distribution of 33 motifs in csmVariants across 33 cancer types.

Motif ‘NCGDHNDHCGN’ was enriched in csmVariants adjacent context. We have identified 35 motifs that were significantly enriched in context sequences of csmVariants (10 flanking bases 5’ and 3’, Figure 2A). To verify the specificity of these enriched motifs, we conducted a motif enrichment analysis on both ncsmVariants and the common SNPs set. The motif ‘NCGDHNDHCGN’ constituted the most frequent occurrence, representing 53.02% of the csmVariants context. Meanwhile, it constituted 9.07% of the ncsmVariants and 11.75% of the common SNPs set (Figure 2A, Supplementary Table S8). However, the frequency of the ‘CCCCGCCCCCMCC’ motif was found to be 1.12%, 6.38% and 9.10% in the csmVariants, ncsmVariants, and common SNPs set, respectively. Furthermore, the frequency of the ‘TTAAAAAAAAG’ motif was observed to be 0.84%, 7.96% and 3.97% in the csmVariants, ncsmVariants and common SNPs set, respectively. The distribution of these 35 enriched motifs across 33 different cancer types was also displayed separately (Figure 2B, Supplementary Table S9). The frequency of these motifs varied between different types of cancers. For example, the top motif observed in TCGA-BRCA was ‘ACAACAAC’ (12.59%), while the motif ‘ACTCTGTCTC’ had the highest frequency in TCGA-GBM (20.72%).

35.93% of csmVariants contexts contained palindromic sequence. Among the csmVariant contexts that supported the ‘NCGDHNDHCGN’ motif, 11.67% of them begin with ‘CG’ and end with ‘CG’, and 7.03% of these sequences were palindromic, such as ‘CGGTAACCG’. 7.43% of csmVariant contexts exhibited a precisely palindromic pattern, and 28.50% displayed a near-palindrome structure with one mismatch (Fisher’s test $P-value < 2.2e-16$, Supplementary Table S10). In contrast, ncsmVariants revealed that 5.53% were precisely palindromic, and 13.97% exhibited a near-palindrome pattern with one mismatch. The prevalence of palindromic sequences within the context of csmVariants suggested that these sequences may significantly contribute to the elevated somatic mutation incidence rates observed in csmVariants.

### Associations of csmVariants rates with clinical features in the TCGA dataset

**Figure 3:**
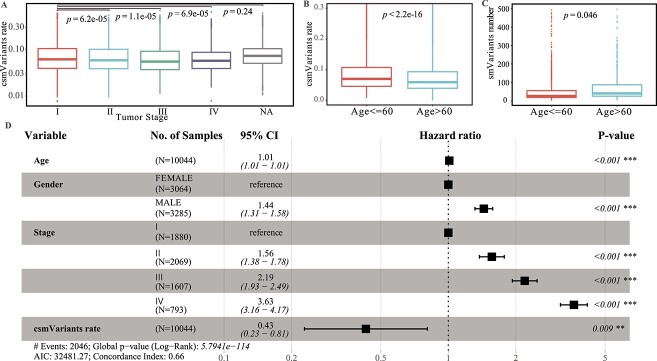
**The associations of csmVariants rate with clinical features.** (A) Comparing the csmVariants rate difference between different tumor stages. The csmVariants rate refers to the percentage of single base csmVariants divided by the total somatic small variants number. (B) Comparing the csmVariants rate difference between the young group ($<=60$)and the elder group ($>60$). (C) Comparing the csmVariants number difference between the young group ($<=60$)and the elder group ($>60$) in TCGA pan-cancer. (D) The PFS forest Plot for multivariate Cox regression by considering age, gender, stage and csmVariants rate.

The csmVariants rate varied according to different cancer types and cancer stages. The csmVariants rate was the percentage of csmVariants divided by total somatic mutations. The TCGA dataset revealed a median csmVariants rate of 6.40% (Supplementary Figure S4A). TCGA-THCA demonstrated the highest csmVariants rate (15.03%), while TCGA-SKCM exhibited the lowest csmVariants rate (2.94%). Tumor samples of stage I had a significantly higher csmVariants rate than samples of other stages II, III and IV (Figure 3A). The higher csmVariants rate in stage I indicated the faster accumulation of csmVariants in the early-stage tumor.

The csmVariants rate was significantly higher in the younger group than in the elder group. The TCGA cohort can be divided into two groups: the elder group ($>60$) and the younger group ($<=60$). The csmVariants rate was significantly higher in the younger group compared with the elder group (mean value: 9.34% versus 7.92%, t.test $P-value < 2.2e-16$, Figure 3B), whereas the elder group had more csmVariants than the younger group (mean value: 55.61 versus 51.58, t.test $P-value = 0.046$, Figure 3C). As the hyper-mutated samples had a higher mutation rate, we excluded samples with more than 500 csmVariants; The elder group still had more csmVariants (mean value: 50.23 versus 43.49, t.test $P-value = 1.64e-09$), and the csmVariants number difference between the older group and the younger group in non-hyper-mutated samples was higher than the difference in total TCGA samples. The fewer csmVariants in the elder group of TCGA-UCEC than the younger group was caused by the enrichment of hyper-mutated samples in the TCGA-UCEC younger group (samples with $csmVariants> 500$ and $age> 60$: 11; samples with $csmVariants> 500$ and $age <= 60$: 23; Supplementary Figure S4B). The elder group had more csmVariants, which indicated aging contributed to the occurrence of csmVariants.

csmVariants rate can serve as a valuable prognostic indicator for cancer patients. Taking age, gender, stage and csmVariants rate into the Cox proportional hazards model, TCGA patients with higher csmVariants rates had better progression-free survival (PFS, hazard ratio = 0.43, log-rank $P-value = 0.009$, Figure 3D), while the TCGA-SKCM patients with higher csmVariants rates had worse overall survival (OS, hazard ratio = 3 760.6, log-rank $P-value < 0.001$, Supplementary Figure S1D). The TCGA-UCEC patients with higher csmVariants rates also had worse OS (hazard ratio = 61, log-rank $P-value = 0.007$, Supplementary Figure S1E, Supplementary Table S11).

### csmVariants with high somatic mutation frequency tend to cluster in cancers with DNA mismatch repair deficiency

**Figure 4:**
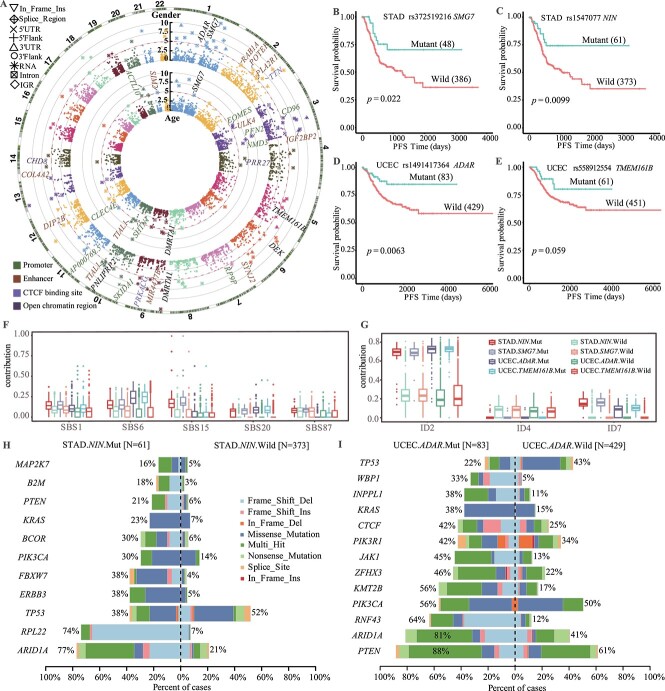
**The clinical associations of the frequently mutated csmVariants.** (A) The inner circle presents the age-related csmVariants, and the outer circle displays the gender-related csmVariants. We marked the gene names of csmVariants located in regulatory regions with $P-value < 0.05 / 4,271$ (the number of csmVariants mutated in more than 15 samples). The PFS curves of TCGA-STAD patients who carried the csmVariants rs372519216 (B) and rs1547077 (C) or not. TCGA-UCEC patients carried csmVariants rs1491417364 (D), and rs558912554 (E) had better PFS. P-value, log-rank test. Comparing the contribution of SBSs (F) and IDs (G) in TCGA-STAD and TCGA-UCEC patients who carried selected csmVariants or not. (H) The mutation frequency difference of SMGs in the TCGA-STAD cohort grouped by csmVariants rs1547077. (I) The mutation frequency difference of SMGs in the TCGA-UCEC cohort grouped by csmVariants rs1491417364.

The csmVariants with high somatic mutation frequency had tendencies to certain age or gender groups. With 4 271 csmVariants (1.25%) detected as somatically mutated in over 15 TCGA tumor samples, we delved into the proclivity of these csmVariants with high somatic mutation frequency. The pattern of csmVariants toward specific groups demonstrated the enrichment of extrinsic and intrinsic mutagenic processes. For each frequently mutated csmVariant, we constructed a 2x2 contingency table and employed Fisher exact test to calculate the significance of the association between csmVariants and age or gender. Our analysis yielded 428 significant age-related csmVariants ($P-value < 0.01$) and 473 significant gender-related csmVariants (csmVariants located on X or Y chromosome were removed; $P-value < 0.01$, Figure 4A). After identifying 38 gender-related functional csmVariants ($P-value < 0.01$), we used DAVID [25] to cluster the genes that carry these csmVariants. These genes were significantly enriched in the ‘Helicase’ molecular function (FDR $q-value = 0.0066$, Supplementary Table S12). In addition, the 32 age-related functional csmVariants were found to be enriched in the ‘Transcription Factor Binding Sites’ biological process based on the genes that contain them (FDR $q-value = 0.066$, Supplementary Table S13).

The csmVariants rs372519216, situated in the intron of *SMG7*, was found to have a higher mutation frequency in the female than the male (113 versus 27) and in the elder group compared with the younger group (100 versus 40). Out of the 140 patient carrying this csmVariant, 67 were diagnosed with UCEC and 48 with Stomach Adenocarcinoma (STAD). The mutation frequency of rs372519216 in TCGA-UCEC and TCGA-STAD was 12.23% and 10.84%, respectively. Notably, in TCGA-STAD, the csmVariant rs372519216 also tended to be somatically altered more in females compared with males (Female versus Male: 30/158 versus 18/285, Fisher’s test $P-value = 8.86e-05$). *SMG7* encodes a p53-binding protein that has a vital role in the regulation of p53-mediated response to DNA damage [26].

These csmVariants with high somatic mutation frequency can serve as valuable biomarkers for molecular classification. We identified 11 csmVariants with mutation frequency higher than 10% in any TCGA cancer type (Supplementary Table S14) and explored the genomic or clinical characteristics of cancer patients who carried these csmVariants. In TCGA-STAD, patients carrying csmVariant rs372519216 or rs1547077 (located in the intron of *NIN*) had significantly better PFS (Figure 4B, C), while the TCGA-UCEC patients with csmVariants rs1491417364 (located in the intron of *ADAR*) or rs558912554 (located in the intron of *TMEM161B*) also exhibited better PFS (Figure 4D, E). The COSMIC database has documented various mutational signatures linked to defective MMR, including SBS6, SBS15, SBS20, ID2, and ID7. These signatures were particularly common in cancer patients with the four csmVariants in TCGA-STAD and TCGA-UCEC (Figure 4F, G). This observation implies that defective MMR plays a significant role as a carcinogenic factor in cancers characterized by these specific csmVariants mutations.

Distinct mutational landscapes were observed in TCGA-STAD and TCGA-UCEC subtypes classified based on the mutation status of these four csmVariants. In TCGA-STAD, the mutant group of rs1547077 or rs372519216 had higher mutation frequency in genes such as *ARID1A, RPL22, ERBB3, FBXW7, BCOR, KRAS, PTEN, B2M* and *MAP2K7* compared with the wild group (Figure 4H, Supplementary Figure S5K). In contrast, in TCGA-UCEC, the mutant group of rs1491417364 or rs558912554 had higher mutation frequency in genes such as *ARID1A, RNF43, KMT2B, ZFHX3, JAK1, CTCF, KRAS, INPPL1* and *WBP1* compared with the wild group (Figure 4G, Supplementary Figure S5L). Interestingly, the well-known tumor suppressor gene, *TP53*, had lower mutation frequency in cancers that carry these csmVariants.

## CONCLUSIONS

Within the human genome, common loci represent a distinctive set of genetic positions harboring variants found in at least 1% of the population. Our study delved into the somatic mutation patterns within the TCGA mutation dataset, identifying a subset known as csmVariants that displayed a higher propensity for mutation than non-common regions. A total of 123 489 recurrent somatic csmVariants were identified, making up 36.14% of all csmVariants within the TCGA somatic mutation dataset—2.59 times that of the ncsmVariants. This robust finding strongly suggested that the occurrence of somatic mutations at common small variant loci was highly improbable by chance alone. Moreover, the common loci exhibited a significantly higher somatic mutation incidence rate compared with non-common sites within the TCGA mutations dataset. These results strongly indicated that common loci were more prone to mutations than non-common loci.

Due to the potential presence of somatic mutations in blood tissues, sequencing data obtained from blood samples in the 1000 Genomes project might contain numerous somatic mutations. The significant recurrence of csmVariants suggested that the common small variants documented in dbSNP may not necessarily originate from parental sources but rather arise *de novo*. These variant loci exhibited a high mutation rate and had the capacity to accumulate mutations in a substantial number of independent individuals.

Mutational signatures refer to characteristic patterns of mutations that were associated with specific mutational processes or DNA damage mechanisms. In this study, we analyzed the contribution of COSMIC signatures to csmVariants and TCGA datasets. Our findings suggested that certain signatures, such as SBS1, SBS87 and DBS7, had significantly higher contributions to csmVariants than total mutations in TCGA datasets. SBS1, characterized predominantly by C-to-T mutations at CpG sites, was initiated by spontaneous or enzymatic deamination of 5-methylcytosine to thymine. This signature has been associated with endogenous mutational processes such as aging and inflammation. The high cosine similarity between the aging signature (SBS1) and 96-trinucleotide spectrums of csmVariants, and the high contribution of SBS1 to csmVariants indicated that aging is the major mutagens of csmVariants. Similarly, SBS87 is predominantly characterized by substitutions at CpG sites and has been linked to thiopurine chemotherapy treatment. The enrichment of DBS7 signatures in csmVariants suggested that certain mechanisms, such as defective DNA mismatch repair, may be more prone to accumulate csmVariants than ncsmVariant mutations. Although aging induces csmVariants and defective DNA mismatch repair decreases the repair of csmVariants, the enrichment of palindromic sequences at csmVariants sites was an important factor for the high somatic mutation incidence rate of common loci. The palindromic sequence can inhibit the DNA mismatch repair process to increase the accumulation of csmVariants.

As the csmVariants burden is positively related to age at diagnosis and the csmVariants rate was higher in tumors with early tumor stage or younger age, the rate of csmVariants accumulation during tumorigenesis was damped. The csmVariants rates differed across different cancer types and different regions. The lower call rate of non-synonymous csmVariants than that of noncoding or synonymous csmVariants implied that non-functional csmVariants were being conserved by natural selection. The study further analyzed the impact of csmVariants rates on patient survival and found that TCGA patients with higher csmVariants rates had better PFS in pan-cancer analysis, while TCGA-SKCM and TCGA-UCEC patients with higher csmVariants rates had worse overall survival. The higher csmVariants rate in patients in the younger group or stage I indicated associations between the early-stage tumor and high csmVariants rate. These may explain the better prognosis of csmVariants.

Cancer patients subtyping by specific csmVariants, including rs372519216, rs1547077, rs1491417364 or rs558912554, had significantly better PFS in TCGA-STAD and TCGA-UCEC. The study also revealed distinct mutational landscapes in TCGA-STAD and TCGA-UCEC subtypes classified on the basis of the mutation status of these csmVariants. Deficient MMR-related mutational signatures significantly contributed to the mutation process in cancer patients who carry these csmVariants. Our study suggested that csmVariants can be a predictive and prognostic biomarker for personalized treatment strategies for cancer patients. These csmVariants also showed a higher frequency of occurrence in certain cancers and/or in certain gender groups within those cancers. This information may provide insights into potential disease mechanisms and could be used to develop personalized treatment strategies based on an individual’s genetic profile.

Overall, our study delved into somatic mutation patterns in the TCGA mutation dataset, pinpointing a subset of somatic mutations termed csmVariants that exhibited a higher predisposition to mutation than non-common regions. Notably, the common loci displayed a significantly elevated somatic mutation incidence rate compared with non-common sites within the TCGA mutations dataset, providing compelling evidence for the heightened susceptibility of common loci to mutations. Then, our investigation sheds light on the correlation of csmVariants with age, gender, tumor stage and their potential impact on cancer prognosis. The identification of specific csmVariants linked to improved PFS holds implications for tailoring personalized therapies for cancer patients. The accumulation of csmVariants induced by aging, defective DNA mismatch repair and palindromic sequences at csmVariant sites presents a novel perspective for understanding the incidence of somatic mutations. However, more research is needed to fully understand the roles of these csmVariants in cancer development and progression.

**Key Points**

- The prevalent genetic sites exhibit elevated incidence of somatic mutations.
- The csmVariants exhibit a prominent prevalence of C-to-T transitions at CpG sites and a noteworthy enrichment of palindromic sequences.
- The mutational signature associated with aging contributed to 13.87% of the csmVariants, which is 4.5 times greater than that observed in ncsmVariants.
- Cancer patients with higher csmVariants rates had better PFS.

## Supplementary Material

Supplementary_fig1_bbae065

Supplementary_fig2_bbae065

Supplementary_fig3_bbae065

Supplementary_fig4_bbae065

Supplementary_fig5_bbae065

Supplementary_File_1_bbae065

csmVariants_supplementary_bbae065

## Data Availability

The csmVariants data identified from the TCGA project have been uploaded online at https://zenodo.org/doi/10.5281/zenodo.10453323. The code of data analysis and visualization can be accessed online at https://zenodo.org/doi/10.5281/zenodo.10429096.

## Author Biographies

**Mengyao Wang** received a Ph.D. degree in computer science from the City University of Hong Kong in 2023. She has served at Fujian Medical University after graduation. Her research interests encompass various aspects of cancer genomics and cancer immunology.

**Shuai Cheng Li** is an associate professort from the City University of Hong Kong. His current research interests include bioinformatics and algorithms. His focus is on omics data analysis and machine learning.

**Bairong Shen** is a professor and executive director at Institutes for Systems Genetics, West China Hospital, Sichuan University. His research interest is biomedical informatics, genetics and medical systems biology.

## References

[1] Abecasis GR, Altshuler D, Auton A, et al. A map of human genome variation from population-scale sequencing. Nature 2010;467(7319):1061–73. 20981092 10.1038/nature09534 PMC3042601

[2] Sherry ST, Ward MH, Kholodov M, et al. dbSNP: the NCBI database of genetic variation. Nucleic Acids Res 2001;29(1):308–11. 11125122 10.1093/nar/29.1.308 PMC29783

[3] Kiezun A, Pulit SL, Francioli LC, et al. Deleterious alleles in the human genome are on average younger than neutral alleles of the same frequency. PLoS Genet 2013;9(2):e1003301. 23468643 10.1371/journal.pgen.1003301 PMC3585140

[4] Zhao Z, Fu YX, Hewett-Emmett D, Boerwinkle E. Investigating single nucleotide polymorphism (SNP) density in the human genome and its implications for molecular evolution. Gene 2003;312:207–13. 12909357 10.1016/s0378-1119(03)00670-x

[5] Barreiro LB, Laval G, Quach H, et al. Natural selection has driven population differentiation in modern humans. Nat Genet 2008;40(3):340–5. 18246066 10.1038/ng.78

[6] Katsonis P, Koire A, Wilson SJ, et al. Single nucleotide variations: biological impact and theoretical interpretation. Protein Sci 2014;23(12):1650–66. 25234433 10.1002/pro.2552 PMC4253807

[7] Reich DE, Schaffner SF, Daly MJ, et al. Human genome sequence variation and the influence of gene history, mutation and recombination. Nat Genet 2002;32(1):135–42. 12161752 10.1038/ng947

[8] Fryxell KJ, Moon WJ. CpG mutation rates in the human genome are highly dependent on local GC content. Mol Biol Evol 2005;22(3):650–8. 15537806 10.1093/molbev/msi043

[9] Duncan BK, Miller JH. Mutagenic deamination of cytosine residues in DNA. Nature 1980;287(5782):560–1. 6999365 10.1038/287560a0

[10] Bulmer M . Neighboring base effects on substitution rates in pseudogenes. Mol Biol Evol 1986;3(4):322–9. 3444408 10.1093/oxfordjournals.molbev.a040401

[11] Sved J, Bird A. The expected equilibrium of the CpG dinucleotide in vertebrate genomes under a mutation model. Proc Natl Acad Sci U S A 1990;87(12):4692–6. 2352943 10.1073/pnas.87.12.4692 PMC54183

[12] M C, Svetec IK. Palindromes in DNA-A risk for genome stability and implications in cancer. Int J Mol Sci 2021;22(6). 10.3390/ijms22062840 PMC7999016 33799581

[13] Nag DK, White MA, Petes TD. Palindromic sequences in heteroduplex DNA inhibit mismatch repair in yeast. Nature 1989;340(6231):318–20. 2546083 10.1038/340318a0

[14] Balmain A . The critical roles of somatic mutations and environmental tumor-promoting agents in cancer risk. Nat Genet 2020;52(11):1139–43. 33106632 10.1038/s41588-020-00727-5 PMC8360498

[15] Juul M, Bertl J, Guo Q, et al. Non-coding cancer driver candidates identified with a sample- and position-specific model of the somatic mutation rate. Elife 2017;6. 10.7554/eLife.21778 PMC5440169 28362259

[16] Jung H, Bleazard T, Lee J, Hong D. Systematic investigation of cancer-associated somatic point mutations in SNP databases. Nat Biotechnol 2013;31(9):787–9. 24022151 10.1038/nbt.2681

[17] Wang S, Li H, Song M, et al. Copy number signature analysis tool and its application in prostate cancer reveals distinct mutational processes and clinical outcomes. PLoS Genet 2021;17(5):e1009557. 33945534 10.1371/journal.pgen.1009557 PMC8121287

[18] Liu J, Lichtenberg T, Hoadley KA, et al. An integrated TCGA pan-cancer clinical data resource to drive high-quality survival outcome analytics. Cell 2018;173(2):400–16. 29625055 10.1016/j.cell.2018.02.052 PMC6066282

[19] Martincorena I, Raine KM, Gerstung M, et al. Universal patterns of selection in cancer and somatic tissues. Cell 2017;171(5):1029–1041.e21. 29056346 10.1016/j.cell.2017.09.042 PMC5720395

[20] Bailey TL . STREME: accurate and versatile sequence motif discovery. Bioinformatics 2021;37(18):2834–40. 33760053 10.1093/bioinformatics/btab203 PMC8479671

[21] Bailey TL, Grant CE. SEA: simple enrichment analysis of motifs. 2021. Preprint at. 10.1101/2021.08.23.457422.

[22] Alamro H, Alzamel M, Iliopoulos CS, et al. IUPACpal: efficient identification of inverted repeats in IUPAC-encoded DNA sequences. BMC Bioinformatics 2021;22(1):51. 33549041 10.1186/s12859-021-03983-2 PMC7866733

[23] Rodriguez-Galindo M, Casillas S, Weghorn D, Barbadilla A. Germline de novo mutation rates on exons versus introns in humans. Nat Commun 2020;11(1):3304. 32620809 10.1038/s41467-020-17162-z PMC7334200

[24] Meier B, Volkova NV, Hong Y, et al. Mutational signatures of DNA mismatch repair deficiency in C. Elegans and human cancers. Genome Res 2018;28(5):666–75. 29636374 10.1101/gr.226845.117 PMC5932607

[25] Sherman BT, Hao M, Qiu J, et al. DAVID: a web server for functional enrichment analysis and functional annotation of gene lists (2021 update). Nucleic Acids Res 2022;50(W1):W216–21. 35325185 10.1093/nar/gkac194 PMC9252805

[26] Luo H, Cowen L, Yu G, et al. SMG7 is a critical regulator of p53 stability and function in DNA damage stress response. Cell Discov 2016;2:15042. 27462439 10.1038/celldisc.2015.42 PMC4860962

