# Supplementary material for: Elevated incidence of somatic mutations at prevalent genetic sites
Source: Brief Bioinform. 2024 Feb 28;25(2):bbae065. doi: 10.1093/bib/bbae065 (PMC10939422; doi:10.1093/bib/bbae065)
Supplement: Supplementary_File_1_bbae065 [file supplementary_file_1_bbae065.docx]

Supplementary File 1

Elevated Incidence of Somatic Mutations at Prevalent Genetic Sites

Mengyao Wang^1,2^, Shuai Cheng Li^1,*^, and Bairong Shen^3,*^

^1^ Department of Computer Science, City University of Hong Kong, 83 Tat Chee Ave, Kowloon Tong, Hong Kong, China,

^2^ Key Laboratory of Ministry of Education for Gastrointestinal Cancer, School of Public Health, Fujian Medical University, Fuzhou, Fujian, China
^3^ Institutes for Systems Genetics, Frontiers Science Center for Disease-Related Molecular Network, West China Hospital, Sichuan University, Chengdu, Sichuan, China

^*^ Corresponding authors: Shuai Cheng Li ([shuaicli@cityu.edu.hk](mailto:shuaicli@cityu.edu.hk)) and Bairong Shen (bairong.shen@scu.edu.cn)

**Table of Contents**

[**Figure S1 | The multivariate Cox regression forest plot.** 2](#_Toc155105149)

[**Figure S2 | Comparison of mutational spectrum in csmVariants and ncsmVariants.** 3](#_Toc155105150)

[**Figure S3 | Comparison of DBS and ID mutational signature contributions across different cancer types.** 4](#_Toc155105151)

[**Figure S4 | Overview the total csmVariants number and csmVariants rate in different cancer types.** 5](#_Toc155105152)

[**Figure S5 | The survival curves of frequently mutated csmVariants in TCGA-COAD, TCGA-STAD, and TCGA-UCEC.** 6](#_Toc155105153)


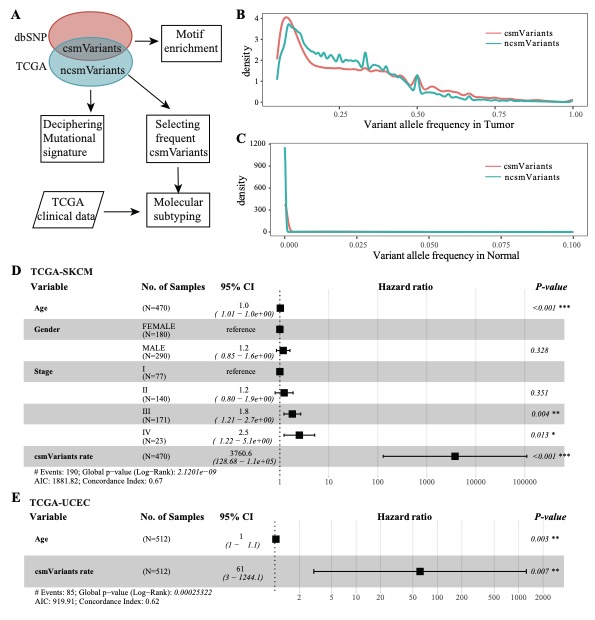


**Figure S1 | The multivariate Cox regression forest plot.**

(A) The analysis pipeline of csmVariant comparison. We compared the variant allele frequency density distribution of csmVariants and TCGA somatic mutations in tumor (B) and normal (C) tissue. (D) The OS forest Plot for multivariate Cox regression by considering age, gender, stage, and csmVariants rate in TCGA-SKCM. (E) The OS forest Plot for multivariate Cox regression by considering age and csmVariants rate in TCGA-UCEC.

**Figure S2 | Comparison of mutational spectrum in csmVariants and ncsmVariants.**

Comparing the mutational spectrums of SBS (A), DBS (B), and ID (C) in common SNPs, ncsmVariants, and csmVariants.

**Figure S3 | Comparison of DBS and ID mutational signature contributions across different cancer types.**

Comparison of DBS (A) and ID (B) mutational signature contributions across different cancer types in csmVariants and ncsmVariants.


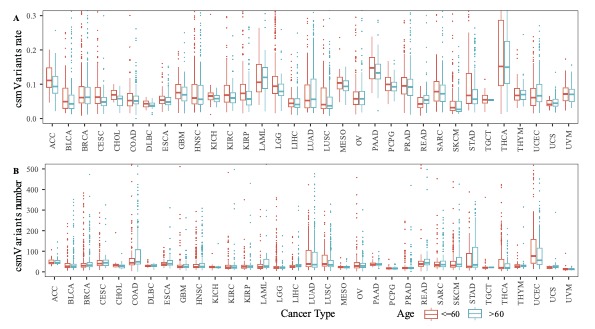


**Figure S4 | Overview the total csmVariants number and csmVariants rate in different cancer types.**

We compared the csmVariants rate (A) and total csmVariants number (B) across different cancer types.


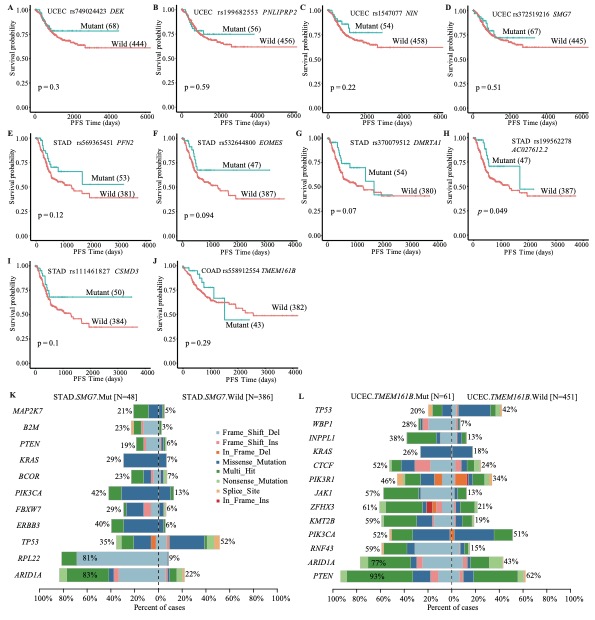


**Figure S5 | The survival curves of frequently mutated csmVariants in TCGA-COAD, TCGA-STAD, and TCGA-UCEC.**

Kaplan-Meier PFS survival analysis of TCGA-UCEC patients stratified by whether they carried csmVariants rs749024423 (A), rs199682553 (B), rs1547077 (C), and rs372519216 (D). Kaplan-Meier PFS survival curves of TCGA-STAD patients stratified by whether they carried csmVariants rs569365451 (E), rs532644800 (F), rs370079512 (G), rs199562278 (H), and rs111461827 (I). (J) The PFS curves of TCGA-COAD patients who carried csmVariant rs558912554 or not. We also compared the mutation frequency of selected SMGs in TCGA-STAD (K) and TCGA-UCEC (L).
